# Analysis of plant growth-promoting properties of *Bacillusamyloliquefaciens* UCMB5113 using *Arabidopsis thaliana* as host plant

**DOI:** 10.1007/s00425-016-2580-9

**Published:** 2016-08-19

**Authors:** Shashidar Asari, Danuše Tarkowská, Jakub Rolčík, Ondřej Novák, David Velázquez Palmero, Sarosh Bejai, Johan Meijer

**Affiliations:** 1 Department of Plant Biology, Uppsala Biocenter, Swedish University of Agricultural Sciences and Linnéan Center for Plant Biology, Box 7080, S75007 Uppsala, Sweden; 2 Laboratory of Growth Regulators, Centre of the Region Haná for Biotechnological and Agricultural Research, Institute of Experimental Botany ASCR and Palacký University, Šlechtitelů 11, CZ-783 71 Olomouc, Czech Republic

**Keywords:** Beneficial bacteria, Biocontrol, Growth promotion, Phytohormones, Rhizosphere, Root structure

## Abstract

**This study showed that** ***Bacillus*** ***amyloliquefaciens*** **UCMB5113 colonizing** ***Arabidopsis*** **roots changed root structure and promoted growth implying the usability of this strain as a novel tool to support sustainable crop production.**

Root architecture plays a crucial role for plants to ensure uptake of water, minerals and nutrients and to provide anchorage in the soil. The root is a dynamic structure with plastic growth and branching depending on the continuous integration of internal and environmental factors. The rhizosphere contains a complex microbiota, where some microbes can colonize plant roots and support growth and stress tolerance. Here, we report that the rhizobacterium *Bacillus amyloliquefaciens* subsp. *plantarum* UCMB5113 stimulated the growth of *Arabidopsis* *thaliana* Col-0 by increased lateral root outgrowth and elongation and root-hair formation, although primary root elongation was inhibited. In addition, the growth of the above ground tissues was stimulated by UCMB5113. Specific hormone reporter gene lines were tested which suggested a role for at least auxin and cytokinin signaling during rhizobacterial modulation of *Arabidopsis* root architecture. UCMB5113 produced cytokinins and indole-3-acetic acid, and the formation of the latter was stimulated by root exudates and tryptophan. The plant growth promotion effect by UCMB5113 did not appear to depend on jasmonic acid in contrast to the disease suppression effect in plants. UCMB5113 exudates inhibited primary root growth, while a semi-purified lipopeptide fraction did not and resulted in the overall growth promotion indicating an interplay of many different bacterial compounds that affect the root growth of the host plant. This study illustrates that beneficial microbes interact with plants in root development via classic and novel signals.

**Electronic supplementary material:**

The online version of this article (doi:10.1007/s00425-016-2580-9) contains supplementary material, which is available to authorized users.

## Introduction

Roots support plants physically by anchoring and chemically through the acquisition of nutrients and water from soil. The root architecture adopts the most suitable structure depending on plant needs and is influenced by various environmental stimuli (Rogers and Benfey 2014; Pacifici et al. 2015). The root is a complex organogenic structure, where information from other plant tissues is integrated together with data from probing of the local soil environment in order for the plant to develop an appropriate root architecture (Pacifici et al. 2015; Rogers and Benfey 2014). A wide range of nutrients and signaling molecules are exuded from roots directing the plant–microbe interactions (Badri and Vivanco 2009). Soil houses a complex microbiota, including strains, that can be beneficial or detrimental to plants. Certain organic compounds exuded act as chemical signals for microbes by stimulating colonization through chemotaxis (Alexandre 2010). Many rhizobacteria can thus associate with plants and ideally promote plant growth and fitness and are referred to as plant growth-promoting rhizobacteria (PGPR) (Lucy et al. 2004). The associated microbes may affect root morphogenesis (Persello-Cartieaux et al. 2003), and many studies suggest that the acceleration of plant growth by PGPR involves phytohormone modulation.

Plant development and root architecture are dependent on the level of hormones and nutrients, where PGPR can have a substantial influence (Vacheron et al. 2013). PGPR can support nutrient uptake, e.g., solubilize phosphorous and iron, which can stimulate initiation, elongation, and development of lateral roots (Ortíz-Castro et al. 2009). Many soil microorganisms produce auxins, which include indole-3-acetic acid (IAA) and indole-3-butyric acid that can support plant growth (Martínez-Morales et al. 2003; Spaepen et al. 2007). It has been reported that PGPR producing IAA stimulated growth of various plants, such as sugar beet (Loper and Schroth 1986), *Brassica juncea* (Asghar et al. 2002), and wheat (Khalid et al. 2004). Cytokinins (CKs) are involved in plant cell division, shoot formation, primary root growth, and callus formation and are utilized in shoot and root meristems (Howell et al. 2003). It is known that certain rhizobacteria, such as *Bacillus megaterium,* can produce CKs that stimulate host plants (Ortíz-Castro et al. 2009). Gibberellins (GA) are another group of plant hormones that influence various developmental processes in plants, including seed germination, stem elongation, flowering, sex expression, and fruit formation (Claeys et al. 2014). It has been shown that PGPR may produce GA (Bastian et al. 1998; Gutierrez-Manero et al. 2001; Joo et al. 2004) that stimulate plant growth. Brassinosteroids are steroid hormones that are essential to regulate plant growth and development and have been recognized as one of the major plant hormones (Zhu et al. 2013).

Use of microorganisms as environmental friendly strategies to support crop production has great potential. A lot of attention has been given to microorganisms that confer disease suppression (Choudhary and Johri 2009) referred to as priming of induced systemic resistance (ISR) (Pieterse et al. 2012). We have identified *Bacillus amyloliquefaciens* strains with ability to suppress common *Brassica* fungal phytopathogens (Danielsson et al. 2007; Sarosh et al. 2009) and support abiotic stress tolerance (Abd El-Daim et al. 2014). During such studies, we frequently observed growth stimulation of plants, including oilseed rape, wheat, and *Arabidopsis thaliana*. Microorganisms that both stimulate plant growth and stress management provide a good model system to study the mechanisms supporting these different properties. The aim of this work was to characterize plant growth promotion properties of UCMB5113 using *A.* *thaliana* Col-0 as host plant. Use of an axenic model system allowed studies of effects on root system architecture, where mutants and reporter lines enabled an approach to study the role for some common signals and how this correlated with changes in the root system.

## Materials and methods

### Bacterial growth conditions and plant inoculation

The *Bacillus amyloliquefacienes* subsp. *plantarum* UCMB5113 (UCM, Kiev, Ukraine) was maintained on LB medium. A colony was inoculated in LB and grown overnight at 180 rpm, 28 °C. Next day, the bacterial culture was diluted to 10^7^ cfu ml^−1^. Surface sterilized *Arabidopsis thaliana* Col-0 seeds (NASC, Nottingham, UK) were germinated on 0.2× Murashige-Skoog (MS) medium, including vitamins (MS0222, Duchefa, Netherlands), 0.6 % Bacto agar plates. Later, plates were shifted to a growth chamber at 22 °C with 16/8 h photoperiod. After 5 days, seedlings were transplanted to new square petri dishes containing 0.2× MS, 0.8 % Bacto agar. Aliquots of usually 10 or 25 µl (corresponding to 2 × 10^7^ or 5 × 10^7^ cfu) of *Bacillus* UCMB5113 were inoculated at a distance of 3 cm from the root tip, and plates were placed vertically in a growth chamber for 6 days (four plants in each plate with 2.5 cm inter distance). After 6 days, the root and shoot was separated and fresh and dry biomass determined. The experiments were repeated three times. Bacterial colonization of roots was verified by tissue printing using filters replicas on LB plates and analysis as described (Johansson et al. 2014).

Bacterial exudates were collected from day 2 to 6 of UCMB5113 grown in LB and tested on 1-week-old *A. thaliana* seedlings. Bacterial exudates and/or auxin were dropped on filter paper discs at 3 cm distance from the main root tip. Six days’ post-inoculation images were taken with a digital camera.

### Root architecture

Digital images of petri dishes of *A. thaliana* seedlings (*n* = 16) were taken using a digital camera positioned at the same distance from the samples. The Rhizo software (Armengaud et al. 2009) was used to measure the primary root and the lateral roots. For root-hair analysis, digital images were taken using a stereo microscope and a section of 2 cm from the primary root tip used for analysis by image J (Schneider et al. 2012) to determine the number of root hairs.

### Auxin and CK production by *B. amyloliquefacienes* UCMB5113

Production of IAA by *Bacillus* UCMB5113 was determined as described previously (Glickmann and Dessaux 1995). Bacterial exudates were collected from day 0 to 4 of UCMB5113 grown in LB or *Arabidopsis* root exudates (10 % final concentration) with or without tryptophan (0.2 mg ml^−1^). Production of CKs by *Bacillus* UCMB5113 was determined in bacterial exudates collected from day 0 to 5 of UCMB5113 grown in LB with or without oilseed rape root exudates. Quantitation of CK metabolites was performed according to the modified method described by Antoniadi et al. (2015) based on Svačinová et al. (2012).

### Isolation of root exudates

*Arabidopsis thaliana* Col-0 seeds were surface sterilized and germinated in a 500 ml flask containing 0.5× MS medium at 22 °C, 16/8 h photoperiod with shaking for 10–14 days. The aqueous phase exudates were lyophilized, resuspended in sterile water, and sterile filtered before use.

### Analysis of reporter lines

The expression of *DR5:GFP* and *Arr5:Gus* plants and response to UCMB5113 was studied 1–6 days post-inoculation and using pieces of the primary or lateral root (2 cm from the root tip) that was detached and placed on a glass slide. Fluorescent images were taken at 6 dpi with a Zeiss confocal scanning microscope 780 (Zeiss, Jena, Germany). Green fluorescent protein was excited at 488 nm and detected at 493**–**530 nm. The images were analyzed using the built-in Zen2011 software. *Cyclin1:Gus* (Donnelly et al. 1999) 5-day-old seedlings were inoculated with UCMB5113 and 6 days later analyzed for dividing cells in the proliferation zone of the primary root tip.

### Phytohormone analysis of plant tissues

Five-day-old seedlings were co-cultivated on 0.2× MS agar plate with *Bacillus* UCMB5113 for 6 days. The root and shoot were then separated for quantitative hormone analysis performed according to following protocols. Samples were analyzed for GA content according to Urbanová et al. (2013) with modifications. Root and shoot samples (10 mg fresh weight) were homogenized in 2 ml polypropylene tubes with 1 ml of 80 % (v/v) acetonitrile containing 5 % (v/v) formic acid using an MM 301 mixer mill (Retsch, Haan, Germany) at a frequency of 27 Hz for 3 min after adding 2 mm zirconium oxide beads with 1 ml of 80 % (v/v) acetonitrile containing 5 % (v/v) formic acid and 19 internal GA standards ([^2^H_2_]GA_1_, [^2^H_2_]GA_3_, [^2^H_2_]GA_4_, [^2^H_2_]GA_5_, [^2^H_2_]GA_6_, [^2^H_2_]GA_7_, [^2^H_2_]GA_8_, [^2^H_2_]GA_9_, [^2^H_2_]GA_12_, [^2^H_2_]GA_12_ald, [^2^H_2_]GA_15_, [^2^H_2_]GA_19_, [^2^H_2_]GA_20_, [^2^H_2_]GA_24_, [^2^H_2_]GA_29_, [^2^H_2_]GA_34_, [^2^H_2_]GA_44_, [^2^H_2_]GA_51,_ and [^2^H_2_]GA_53_) (OlChemIm, Olomouc, Czech Republic) The tubes were then placed in a 4 °C fridge and extracted overnight with constant stirring at a frequency of 15 rpm. The homogenates were centrifuged for 10 min at 4 °C. Supernatants were further purified using mixed-mode anion exchange cartridges (Waters, Milford, MA, USA) and analyzed by ultra high-performance chromatography (Acquity UPLC™ System; Waters) coupled to triple-stage quadrupole mass spectrometer (Xevo^®^ TQ MS; Waters) equipped with an electrospray ionization (ESI) interface. GA were detected using the multiple-reaction-monitoring mode based on the transition of the precursor ion [M–H]^−^ to the appropriate product ion. Data were acquired and processed by the Masslynx 4.1 software (Waters), and GA levels were calculated using the standard isotope-dilution method (Rittenberg and Foster 1940).

The auxin analysis has been performed as described earlier by Pěnčík et al. (2009) with some modifications. Briefly, frozen barley seed samples (5 mg of fresh weight) were homogenized in 2 ml polypropylene tubes and extracted for 5 min with 1 ml of cold phosphate buffer (50 mM; pH 7.0) containing 0.02 % sodium diethyldithiocarbamate and [^2^H_5_]IAA as an internal standard. After centrifugation (36,000×*g*; 10 min; +4 °C), each sample was transferred into a new Eppendorf tube, acidified with 1 M HCl to pH 2.7 and subjected to a C8-based solid-phase extraction, methylated with ethereal diazomethane, and subsequently purified by immunoaffinity extraction. The final analysis was done by ultra-HPLC (Acquity UPLC™ System) coupled to tandem mass spectrometer (Xevo^®^ TQ MS) equipped with the electrospray ionization (ESI) interface operating in the positive mode. Data were acquired and processed by the Masslynx 4.1 software, and IAA levels were calculated using the standard isotope-dilution method on the basis of auxin detection in multiple-reaction-monitoring mode.

For quantitative analysis of brassinosteroids, root and shoot samples of 25 and 50 mg fresh weight, respectively, were sonicated for 5 min and extracted overnight with stirring in ice-cold 60 % (w/v) acetonitrile and 30 pmol of [^2^H_3_]brassinolide, [^2^H_3_]castasterone, [^2^H_3_]24-*epi*-brassinolide, and [^2^H_3_]24-*epi*-castasterone as internal standards (OlChemIm). After centrifugation, samples were further purified on polyamide SPE columns (Supelco, Bellefonte, PA, USA) and then analyzed by ultra-HPLC (Acquity UPLC™ System) coupled to tandem mass spectrometer (Xevo^®^ TQ MS). The data were analyzed using the Masslynx 4.1 software, and brassinosteroid content was quantified using the standard isotope-dilution method based on the detection of analytes in multiple-reaction-monitoring mode.

### Test of crude lipopeptide compounds on plant growth and development

Lipopeptide compounds were isolated from *Bacillus* UCMB5113 by a protocol described by Kim et al. (2004). Sterilized seeds were germinated on petri dishes containing 0.5× MS with 0.6 % agar and incubated at 22 °C, 16/8 light and dark photoperiod. One-week-old seedlings were transplanted on 0.5× MS with 0.8 % agar on 10 × 10 cm square plates and four seedlings on each plate with a distance of 2.5 cm between each seedling. The roots were coated with 5 µl of 2.5 ng ml^−1^ isolated lipopeptide compounds, 5 % methanol or water. The plates were incubated at 22 °C, 16/8 light and dark photoperiod and placed vertically. Five days’ post-inoculation of crude lipopeptide compounds, half the batch (*n* = 20), was moved to sterile soil (S-soil, Weibulls plant soil) with one plant per pot, and the remaining plants were allowed to grow further on MS agar plates. Growth was monitored after 10 days of crude lipopeptide compounds treatment of plants on agar plates and 12 days after on soil-transplanted plants. Flowering was recorded after 21 days, and the number of siliques and seed weight analyzed after 48 days on soil-transplanted plants.

### Statistical analysis

Analysis for significance was calculated using ANOVA, all pairs, Tukey–Kramer HSD (*p* < 0.05).

## Results

### Effect of UCMB5113 on plant growth and root system architecture

**Fig. 1:**
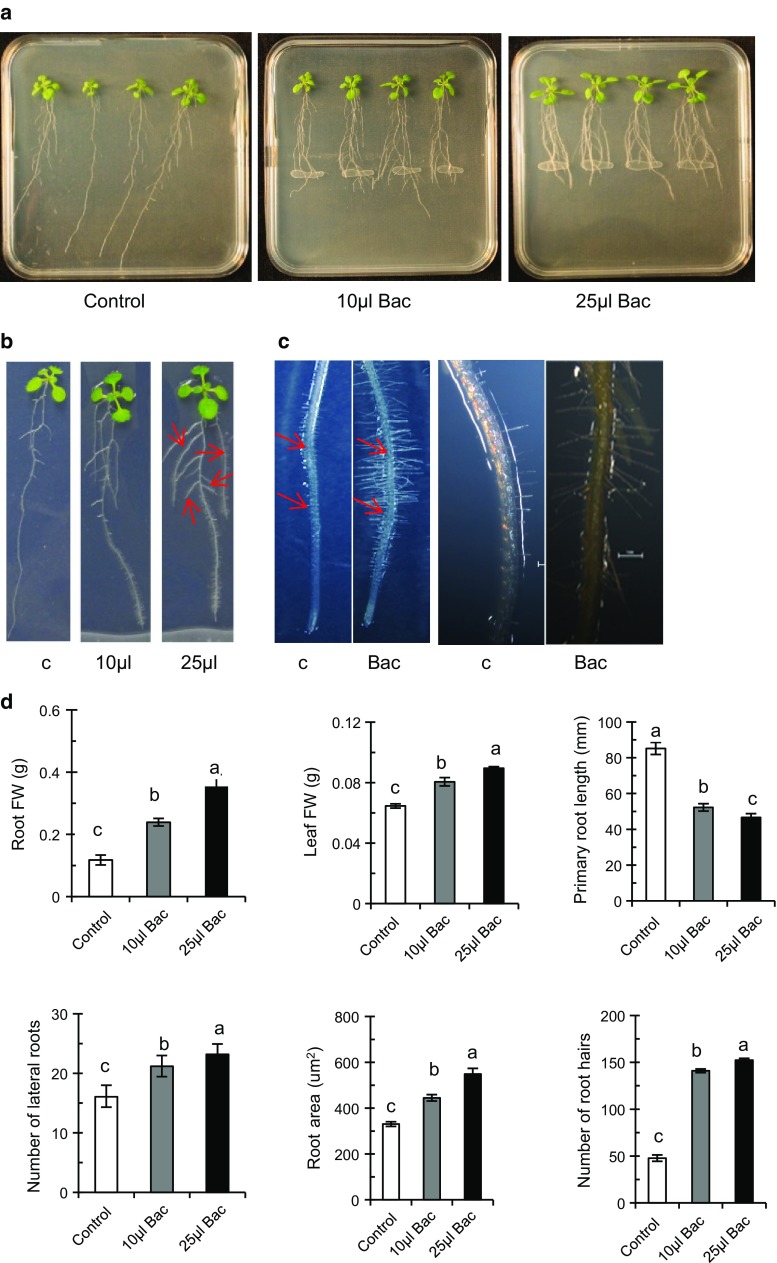
Effects of *Bacillus amyloliquefacienes* UCMB5113 on *A. thaliana* Col-0 seedlings. Five-day-old seedlings were placed vertically on 0.2× MS agar plates with 2.5 cm distance in between plants and treated with water, 10 µl (2 × 10^7^ cfu) or 25 µl (5 × 10^7^ cfu) UCMB5113 as a streak at 3 cm distance from the primary root of the seedlings. After 6 days of co-cultivation, the growth pattern of the whole root was analyzed. Overview of the growth promotion aspect of *Bacillus* treated plants (**a**), closeup pictures, where *arrows* indicate increased number of lateral roots (**b**) and the increased frequency of root hairs on primary and later roots and initiation of root hairs close to root tip after UCMB5113 inoculation (**c**). Root and shoot fresh weight and image analysis of primary root length, total number of lateral roots, and total root area carried out by the rhizo software (**d**). Values represent means and standard deviations (*n* = 16), where samples labelled with identical *letters* are not significant at *p* < 0.05

The effect of plant growth-regulating substances and signaling mechanisms operating as a result of UCMB5113 colonization of *A. thaliana* Col-0 roots was tested in an axenic test system. *Arabidopsis* seedlings were grown vertically on 0.2× MS agar medium. Six days after inoculation with UCMB5113 characteristic changes in root architecture were observed with reorganized overall structure and growth avoidance through the bacterial zone (Fig. 1a–d). Significant increases in fresh weight of plant root and shoot were observed (Fig. 1d). The weight increase was dose dependent, rising 1.5- and 3-folds, and 0.2- and 0.5-folds in the presence of 10 and 25 µl UCMB5113 for root and shoot, respectively. Interestingly, UCMB5113 had a profound effect on the root system architecture and caused almost a 50 % reduction of primary root length but increased outgrowth and elongation of lateral roots, root hairs, and total root area compared to control plants (Fig. 1a–d). UCMB5113 does not seem to interfere with gravitropism, since turning the plate 90° resulted in a gravitropic response of root tips, even though slightly retarded for the inoculated primary root tip. In addition, if plants were inoculated with UCMB5113 alongside with the root and after 1–3 days turned 90°, a gravitropic response was observed (results not shown). If not growth arrest of the primary root occurred, the root tip frequently seemed to grow either below bacteria into the agar or above in the air. The effect of bacterial dosage on plant growth parameters on *Arabidopsis* varied. For example, inoculation with 25 µl UCMB5113 increased root fresh weight compared to 10 µl of UCMB5113 inoculation, while the difference was smaller for lateral root formation, elongation, root-hair number, and root area (Fig. 1d).

Furthermore, we investigated the alteration of cell growth and cell proliferative zones in the primary root after inoculation with UCMB5113. The growth zone on the root tip consists of small dividing cells forming the meristem with more enlarged elongated cells differentiating into other cell types (Gaillochet et al. 2015). Similar overall cell organization of the root tip was observed in both UCMB5113 inoculated and control plants. However, the localization of the cell differentiation zone was closer to the root meristem region with the initiation of root hairs in inoculated plants compared to control plants (Fig. 1c). The analysis of *cyclin1:Gus* plants to measure the number of dividing cells in the primary root division zone found 45 ± 26 and 35 ± 16 cells (*n* = 25) for control and UCMB5113-treated seedlings, respectively. The difference was not statistically significant (*t* test, *p* = 0.112, *df* = 39).

**Fig. 2:**
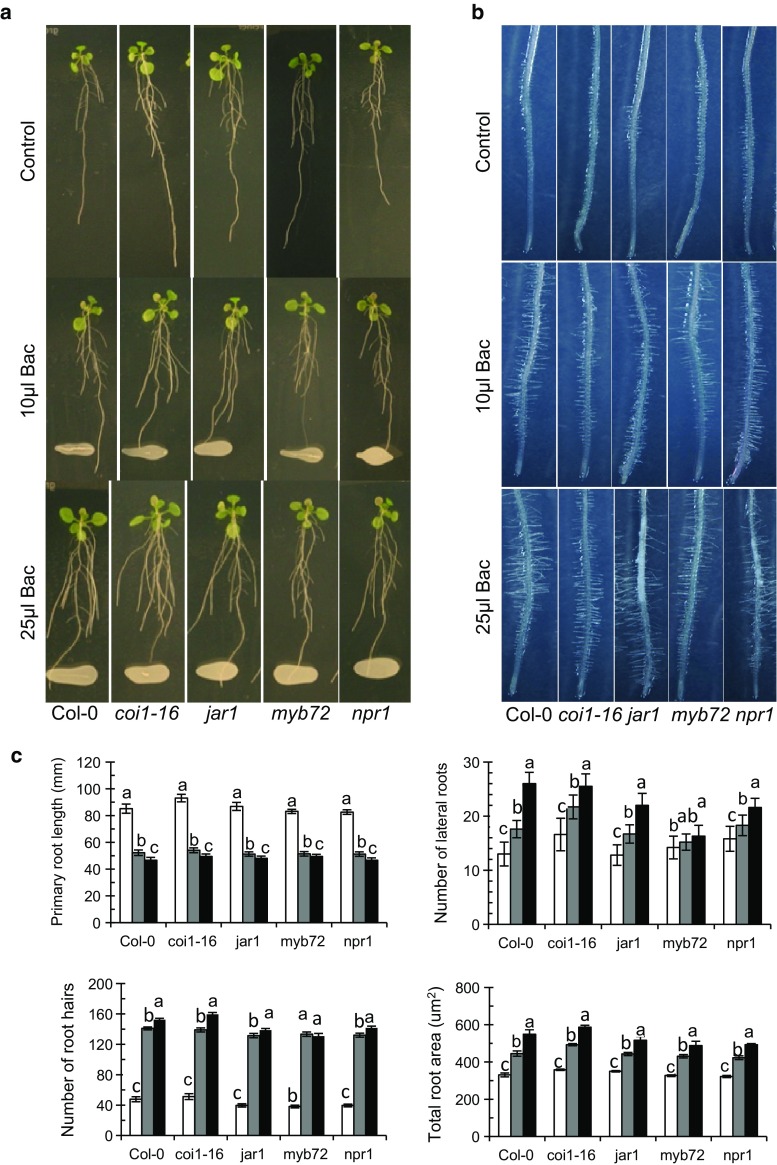
Effects of *Bacillus* UCMB5113 on plant growth of some *Arabidopsis* signaling mutants. Five-day-old seedlings (*n* = 16) were lined up on 0.2× MS agar plates and treated with water or *Bacillus* UCMB5113 (3 cm distance from the main root tip). Six days later digital images were taken for the overall root analysis. **a** Overall *pictures* of water control, 10 or 25 µl UCMB5113-treated plants. **b** Root hairs of control, 10 or 25 µl UCMB5113-treated plants. **c** Image analysis of control (*white bars*), 10 µl (*grey bars*) or 25 µl UCMB5113 (*black bars*) treated plants. Rhizo software was used to determine the primary and lateral root elongation, while root-hair number and total root area were determined with Image J. Values represent means and standard deviations (*n* = 16), where samples with the *same letter* are not significantly different at *p* < 0.05

From other studies, we got indications that UCMB5113 improved stress management involves plant jasmonic acid (JA) signaling. To elucidate the role of JA-dependent plant signaling for the growth promotion activity and altered plant development caused by UCMB5113, we used the signaling mutants *coi1*-*16*, *jar1*, *myb72*, and *npr1*. The overall root morphology was fairly similar among the different plants tested (Fig. 2a). However, the pattern of lateral root outgrowth and root hairs varied among the mutants (Fig. 2a, b). After 6 days of growth in the presence of UCMB5113, the primary root was arrested to 50 % of control value for all plants, but UCMB5113 stimulated lateral roots, root hairs, and root area in all mutants as well as in wild-type plants (Fig. 2c).

### The effect of UCMB5113 on plant phytohormones

**Fig. 3:**
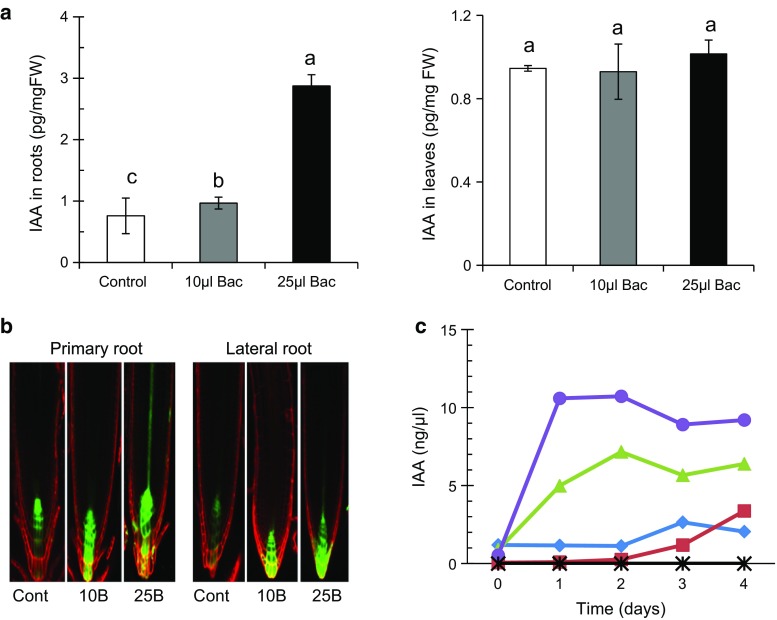
Effect of auxin level in *Arabidopsis* root by UCMB5113 and culture filtrate. Five-day-old seedlings on 0.2× MS agar plates were treated with water or *Bacillus* UCMB5113 and analyzed 6 days later. **a** Auxin analysis of indole-3-acetic acid (IAA) level in *Arabidopsis* roots and shoots. **b** Expression of auxin responsive *DR5:GFP* in primary roots or lateral roots. **c** Production of auxin by UCMB5113 monitored from day 0 to 4 in LB (*diamonds*) supplemented with tryptophan (*squares*), or root exudates (*triangles*) with tryptophan (*circles*), or tryptophan and root exudates but without UCMB5113 (X). Results shown as mean and standard deviation of three samples

Due to the effects on root architectures observed by UCMB5113 inoculation, it was relevant to test to which extent UCMB5113 affected plant growth-regulating substances, including phytohormones, such as auxins, GA, CKs and brassinosteroids. The expression and level of auxin were analyzed in roots and leaves of *A. thaliana* Col-0 plants after 6 days of bacterial inoculation. In plants inoculated with 25 µl UCMB5113, a significant fourfold increase of auxin in roots was observed compared to 10 µl inoculated and control plants (Fig. 3a). However, similar levels of auxin were seen in leaves of inoculated and control plants (Fig. 3a). Furthermore, we examined the auxin expression in *DR5:GFP* *Arabidopsis* auxin response reporter plants. After 6 days of co-cultivation, high expression of *DR5* in the primary root tip and in lateral root meristem cells was observed in 25 µl inoculated plants but less in 10 µl inoculated and only in the zone of quiescent center of control plants (Fig. 3b). To study the potential production of auxin by UCMB5113, the bacteria were grown in LB or root exudates and the effect of supplementation with l-tryptophan was also examined. An increase of auxin production by UCMB5113 was observed from day 1 to 4 in the presence of root exudates, where the addition of tryptophan to the exudate further increased the levels of IAA (Fig. 3c). The amount of auxin remained at a similar level throughout the experimental period. Auxin production was also observed in the absence of root exudates for UCMB5113 grown in LB with or without tryptophan, but the production of auxin was much lower and remained at a low level and without a stimulatory effect by tryptophan as observed for root exudates.

To analyze the effect of UCMB5113 on the levels of GAs in plants, *Arabidopsis* Col-0 root and leaves were analyzed separately after co-cultivation with UCMB5113 for 6 days. No significant difference of total endogenous GAs was observed in roots with only moderate tendency to increase in the presence of bacteria (Table 1). In contrast, the total GAs’ content in leaves was reduced in UCMB5113 inoculated plants compared to untreated plants (Table 2). However, the level of different GA biosynthetic precursors, bioactive GAs, and their metabolite products varied in the presence or absence of UCMB5113 (Tables 1, 2).

**Table 1:** Gibberellin analysis of *Arabidopsis thaliana* Col-0 roots. Gibberellin levels were determined after 6 days of co-cultivation with UCMB5113

Gibberellin	Gibberellin content (pg/mg of root fresh weight)		
	Control	Inoculation (dose dependent)	
		10 μl	25 μl
GAi	0.17 ± 0.02^b^	0.31 ± 0.02^a^	0.15 ± 0.02^b^
GA_3_	0.78 ± 0.16^a^	0.39 ± 0.14^a^	0.66 ± 0.09^a^
GA_4_	0.016 ± 0.007^a^	0.004 ± 0.001^a^	0.008 ± 0.002^a^
GA_5_	6.23 ± 0.74^a^	4.97 ± 0.74^a^	4.64 ± 0.89^a^
GA_6_	0.02 ± 0.01^a^	0.05 ± 0.02^a^	0.05 ± 0.02^a^
GA_7_	0.23 ± 0.02^a^	0.03 ± 0.00^b^	0.05 ± 0.01^b^
GA_8_	1.53 ± 0.16^a^	1.81 ± 0.19^a^	0.88 ± 0.04^b^
GA_9_	1.55 ± 0.28^a^	2.02 ± 0.25^a^	2.29 ± 0.45^a^
GA13	0.07 ± 0.02^a^	0.15 ± 0.01^a^	0.04 ± 0.01^a^
GAi_5_	4.99 ± 0.71^b^	5.73 ± 0.46^b^	17.3 ± 1.79^a^
GA19	0.25 ± 0.04^b^	0.20 ± 0.02^b^	1.31 ± 0.25^a^
GA20	0.64 ± 0.05^a^	0.83 ± 0.10^a^	0.25 ± 0.01^b^
GA_24_	1.28 ± 0.25^b^	2.47 ± 0.33^a^	2.61 ± 0.22^a^
GA29	6.42 ± 0.46^b^	0.72 ± 0.15^a^	2.28 ± 0.07^a^
GA34	0.23 ± 0.13^b^	0.77 ± 0.06^a^	0.77 ± 0.20^a^
GA44	0.88 ± 0.17^a^	1.34 ± 0.06^a^	1.36 ± 0.24^a^
GA_5_i	18.17 ± 1.09^a^	24.15 ± 3.44^a^	19.59 ± 1.82^a^
GA_53_	5.10 ± 1.02^a^	2.91 ± 0.33^a^	0.46 ± 0.06^a^
Total	48.6	48.9	54.6

Treatments labelled with identical letters are not significant at *p* < 0.05

**Table 2:** Gibberellin analysis of *Arabidopsis thaliana* Col-0 shoots. Gibberellin levels were determined after 6 days of co-cultivation with UCMB5113

Gibberellin	Gibberellin content (pg/mg of leaf fresh weight)		
	Control	Inoculation (dose dependent)	
		10 μl	25 μl
GAi	0.14 ± 0.01^b^	0.40 ± 0.10^a^	0.17 ± 0.06^b^
GA_3_	0.68 ± 0.14^a^	0.87 ± 0.11^a^	0.82 ± 0.10^a^
GA_4_	0.0046 ± 0.0008^a^	0.0118 ± 0.0047^a^	0.0184 ± 0.0073^a^
GA_5_	4.28 ± 0.74^a^	4.12 ± 0.21a	1.68 ± 0.23^b^
GA_6_	0.031 ± 0.01 l^ab^	0.033 ± 0.005^ab^	0.014 ± 0.002^b^
GA_7_	0.15 ± 0.03^a^	0.13 ± 0.02^a^	0.10 ± 0.01^a^
GA_8_	1.58 ± 0.21^b^	3.39 ± 0.18^a^	1.24 ± 0.05^b^
GA_9_	1.56 ± 0.27^a^	0.70 ± 0.07^b^	0.40 ± 0.09^b^
GA13	0.038 ± 0.003^b^	0.045 ± 0.005^b^	0.236 ± 0.005^a^
GAi_5_	6.59 ± 0.89^b^	16.57 ± 1.04^a^	2.65 ± 0.09^c^
GA19	0.56 ± 0.03^a^	0.18 ± 0.03^b^	0.33 ± 0.03^c^
GA20	1.03 ± 0.04^a^	0.98 ± 0.13^a^	0.11 ± 0.01^b^
GA_24_	2.50 ± 0.28^a^	1.14 ± 0.12^b^	1.25 ± 0.14^b^
GA29	1.31 ± 0.27^a^	0.62 ± 0.27^b^	1.24 ± 0.07^ab^
GA34	0.73 ± 0.25^a^	0.63 ± 0.09^ab^	0.23 ± 0.04^b^
GA44	0.78 ± 0.06^c^	1.08 ± 0.10^b^	1.87 ± 0.05^a^
GA_5_i	18.04 ± 0.90^a^	22.45 ± 0.47^c^	10.46 ± 0.91^b^
GA_53_	1.69 ± 0.23^b^	2.51 ± 0.17^a^	0.21 ± 0.01^c^
Total	41.7	55.9	23.0

Treatments labelled with identical letters are not significant at *p* < 0.05

**Fig. 4:**
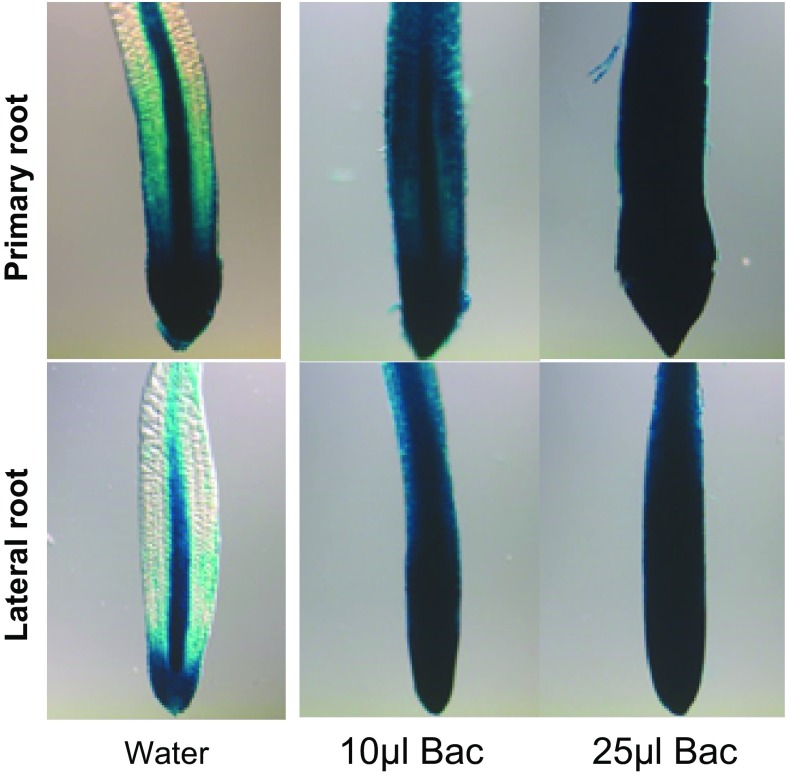
Root expression of the cytokinin responsive reporter *Arr5:Gus*. Five-day-old seedlings on 0.2× MS agar plates were treated with water or *Bacillus* UCMB5113 and analyzed 6 days later for expression of *Arr5:Gus* in primary or lateral roots

Furthermore, we analyzed the potential changes in expression of CKs in planta using *Arr5:Gus* *Arabidopsis* lines as CK responsive and specific reporter plants (D’Agostino et al. 2000). After 6 days of co-cultivation, high expression of *Arr5* in the primary and lateral root was observed in 10 and 25 µl inoculated plants compared to the control indicative of elevated CK levels after UCMB5113 treatment (Fig. 4). To study the potential production of CKs by UCMB5113, the bacteria were grown in LB with or without root exudates. The LB medium contained many CKs, while the root exudate contained much less (Table 3). The presence of UCMB5113 rapidly increased the levels of CK bases and nucleotides, while the levels of CK ribosides and *O*-glucosides rapidly declined (Table 3). Effects on other CKs were small (for example, decreased levels of *N*-glucosides, results not shown). The effects of UCMB5113 on some *Arabidopsis* CK-signaling mutants were tested to study the role of exogenous CK production on plants. Increased root branching and leaf tissue was observed when *arr1* (deficient response regulator that acts in concert with other response regulators in CK signaling) and *cin1* (defective in the induction of ethylene biosynthesis by CK) plants was grown in the presence of UCMB 5113 indicating some complementary effect (results not shown).

**Table 3:** Cytokinin levels in UCMB 5113 exudates

Sample/time	Cytokinin content (fmol/μl)			
	CK-Ba	CK-R	CK-Nuc	CK-Ogl
LB+5113				
T0	16.08 ± 1.24^c^	279.8 ± 32.0^a^	0.61 ± 0.16^b^	17.31 ± 1.87^a^
T24	146.4 ± 17.8^b^	5.46 ± 1.19^b^	3.42 ± 0.63^a^	0.38 ± 0.06^b^
T72	194.7 ± 13.l^a^	1.40 ± 0.26^b^	0.73 ± 0.23^b^	0.32 ± 0.07^b^
T120	152.4 ± 16.9^b^	3.91 ± 0.99^b^	0.63 ± 0.13^b^	0.25 ± 0.02^b^
LB+5113 + root exudate				
T0	15.98 ± 1.08^c^	300.6 ± 29.1^a^	0.66 ± 0.15^b^	16.66 ± 3.65^a^
T24	167.0 ± 13.9^b^	9.45 ± 1.22^b^	1.80 ± 0.29^a^	0.21 ± 0.02^b^
T72	214.9 ± 22.7^a^	1.92 ± 0.43^b^	0.68 ± 0.16^b^	0.10 ± 0.05^b^
T120	205.4 ± 25.9^a^	3.08 ± 0.86^b^	0.39 ± 0.04^c^	0.06 ± 0.02^b^
LB+root exudate				
	14.01 ± 1.64	292.4 ± 43.9	0.53 ± 0.10	18.66 ± 3.36
LB				
	12.91 ± 1.88	273.7 ± 40.4	0.50 ± 0.13	14.94 ± 3.20

Total cytokinin bases (CK-Ba), cytokinin ribosides (CK-R), cytokinin nucleotides (CK-Nuc), and cytokinin O-glucosides (CK-Ogl) were measured in UCMB 5113 exudates at 0 (T0), 24 (T24), 72 (T72), and 120 h (T120) of continuous growth. Means and standard deviation of three biological samples and of each three technical replicates are shown

Treatments labelled with identical letters are not significant at *p* < 0.05

**Fig. 5:**
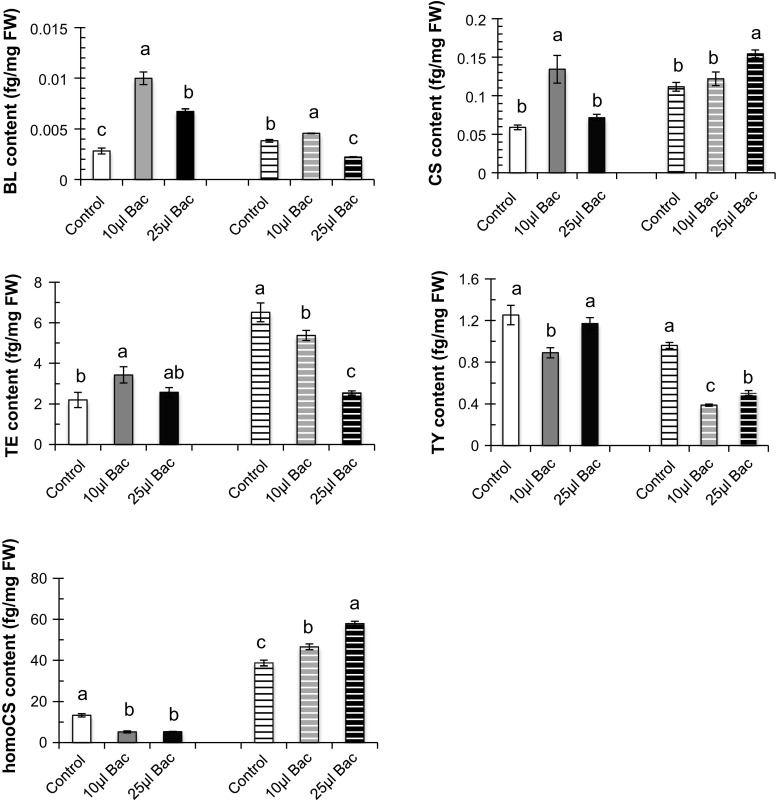
Brassinosteroid analysis of UCMB5113 treated *Arabidopsis*. Five-day-old seedlings on 0.2× MS agar plates were treated with water or *Bacillus* UCMB5113 and analyzed 6 days later for brassinosteroid levels in roots and shoots (*striped bars*). The brassinosteroids analyzed were brassinolide (BL), castasterone (CS), teasterone (TE), typhasterol (TY), and homocastasterone (homoCS). Values represent means and standard deviations (*n* = 4), where samples labelled with *identical letters* are not significant at *p* < 0.05

To investigate whether UCMB5113 inoculation could involve the regulation of steroid hormones in plant development and physiology, brassinosteroids were analyzed. After 6 days of growth in the presence of UCMB5113, the *Arabidopsis* shoot and root were analyzed (Fig. 5). In roots, brassinolide, castasterone, and teasterone levels were highest in 10 µl UCMB5113-inoculated plants, while in leaves, the levels of castasterone and homocastasterone were highest for the 25 µl inoculated plants, respectively. However, the level of different brassinosteroids in roots and shoot varied among the UCMB5113 treatments (Fig. 5).

### Effect of bacterial exudates on root architecture of *Arabidopsis* Col-0

**Fig. 6:**
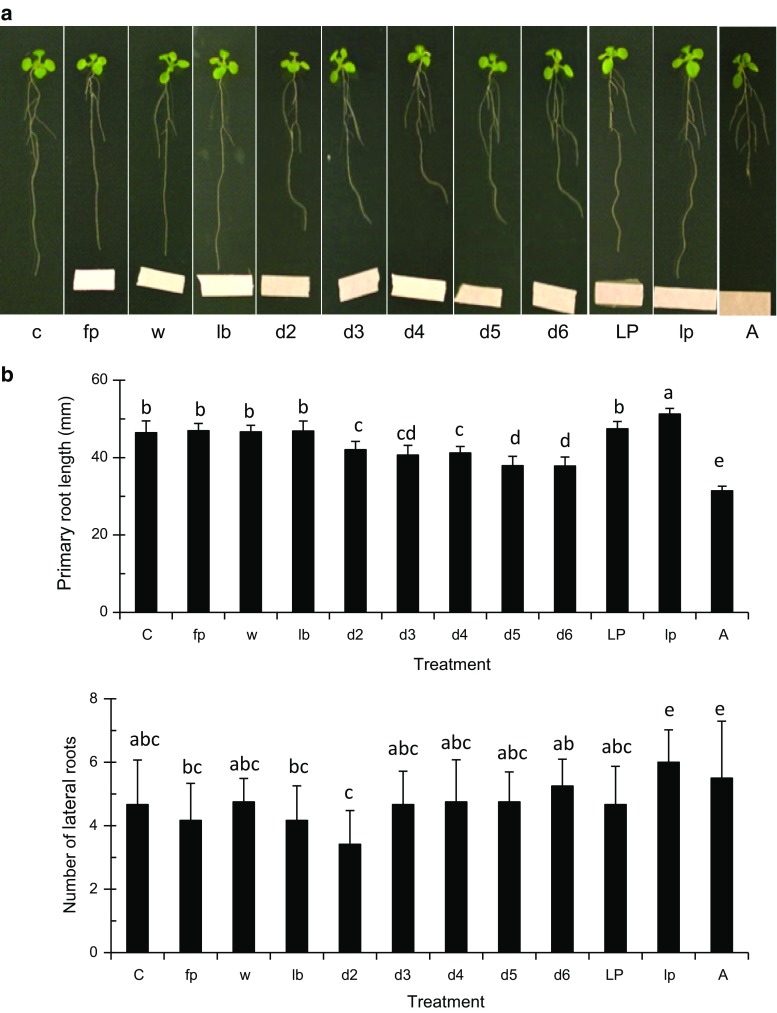
Effect of *Bacillus* compounds on growth and development of *A. thaliana* Col-0. **a** One-week-old *Arabidopsis* seedlings were lined up on 0.2× MS agar plates. *Bacillus* UCMB5113 sterile filtrated culture supernatant from day 2 to 6, crude lipopeptide fraction, or auxin were dropped on filter paper at 3 cm distance from the main root tip. At 6 days of post-inoculation, digital images were taken for the overall root analysis. Samples were control (c), filter paper (fp), 25 µl volume of water (w), LB (lb), culture supernatants from day 2 (d2), day 3 (d3), day 4 (d4), day 5 (d5), day 6 (d6), crude lipopeptides (LP), 10× diluted crude lipopeptides (lp) synthetic auxin applied as 800 pg (A). **b** Analysis of primary root length and total number of later roots deduced by use of the rhizo software. Values represent means and standard deviations (*n* = 16), where samples labelled with *identical letters* are not significant at *p* < 0.05

To further investigate the inhibition of the primary root growth observed after UCMB5113 inoculation, bacterial exudates were tested for their effects. Whole exudates as well as a lipopeptide enriched fraction were tested for their effects to approach the role of bacterial secondary metabolites for growth inhibition. Six-day-old *Arabidopsis* Col-0 seedlings were kept on 0.2× MS agar plates and inoculated at 3 cm distance from the main root with bacterial exudates collected from days 2 to 6 of growth and a crude lipopeptide extract. It was observed that the root increasingly avoided the bacterial exudate from day 2 to 6, but not the lipopeptide extract, although the effect was weaker compared to when the whole bacteria were used (Fig. 6a, b). The effects on lateral roots and root area were also much smaller or insignificant compared to when bacteria were used. Primary root growth retardation was also observed with auxin treatments (Fig. 6a, b) suggesting that the effects are not related to toxic secondary metabolites, but rather hormonal in nature. Fitness effects by lipopeptide treatment of *Arabidopsis* Col-0 were measured as seed parameters, where the number of siliques, seed size, and seed weight increased slightly and even more by a repeated lipopeptide treatment (Supplementary Fig. S1).

### Effect of exudate lipopeptide extract on growth of different *Arabidopsis* lines

**Fig. 7:**
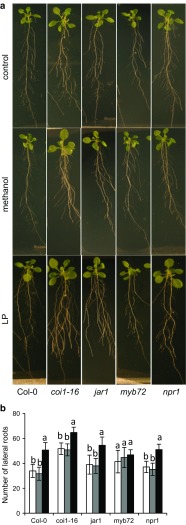
Effect of crude lipopeptide compounds on growth and development of *A*. *thaliana* Col-0 wild type and mutants. One-week-old seedlings of *A*. *thaliana* Col-0 and the mutants *coi1*-*16*, *jar1*, *myb72,* and *npr1* were treated on the roots with water, 5 % methanol (solvent control), or enriched lipopeptide fraction (LP) and grown on 0.5× MS agar plates. Plants were analyzed after 10 days (**a**) and effects on total number of lateral roots measured after treatment with water (*white bars*), methanol (*grey bars*), or enriched lipopeptide fraction (LP) (*black bars*) (**b**). Values represent means and standard deviations (*n* = 16), where samples labelled with *identical letters* are not significant at *p* < 0.05

Lipopeptides have been suggested to prime ISR through JA signaling in other systems. To test effects on plant growth of the lipopeptide extract made from the *Bacillus* exudate, roots of *Arabidopsis* plants were treated with the extract and grown on 0.5× MS agar plates. Crude-extract-treated *Arabidopsis* wild-type (Col-0) plants showed both larger leaves and increased root branching compared to the controls, while no effects on primary roots were observed (Fig. 7a). We then compared the effects on growth in signaling mutants, where *coi1*-*16* and *npr1*-*1* showed similar growth promotion as the wild-type plants. While the *jar1* mutant showed increased root branching (Fig. 7a, b), no effects on leaves occurred compared to plants treated with methanol or water (Fig. 7a). We further tested the growth promotion ability on the above ground tissues of plants grown on soil. Similar results were observed for the leaf size of soil grown wild-type (Col-0), *coi1*-*16* and *npr1*-*1* plants compared to the MS agar grown plants (Supplementary Fig. S2). The time of flowering varied among the treatments and mutants. Compared to the wild type, *coi1*-*16* and *jar1* plants showed earlier bolting and the plants were often taller compared to water and methanol treatment (Supplementary Fig. S3). In the case of *npr1*, *Bacillus* treatment resulted in shorter but flowering plants.

## Discussion

The rhizosphere is abundant with microorganisms that can strongly influence plant performance, for example, by modulating nutrient uptake and thereby either enhance or decrease nutrient availability. Although many studies have been concerned with the beneficial plant–microbe interaction, relatively, few have provided a more thorough description of the effects on the plant by the interaction when it involves PGPR (e.g., Desbrosses et al. 2009; Zamioudis et al. 2013). An inherent difficulty is to study the root architecture of plants that grow in soil. In this study, we used axenically grown *Arabidopsis* as a model system to investigate the effect on phenotypic properties, plant growth regulation, and root architecture by *B. amyloliquefacienes* UCMB5113 known to provide disease suppression (Danielsson et al. 2007; Sarosh et al. 2009).

A positive effect was observed with growth promotion for *Arabidopsis* after UCMB5113 treatment. After inoculation of bacteria, the growth of primary roots was similar in control and inoculated plants during the first 2 days. However, after 3 days of inoculation, a gradual reduction of primary root length but increased outgrowth and elongation of lateral roots was observed in inoculated plants as compared with control plants. This suggests that bacterial metabolites alter the root system and development of the plant by reducing the proliferative activity and growth of the primary root while stimulating premature differentiation of root hairs and lateral root formation. The analysis of cell division in primary root tips using a *cyclin:Gus* reporter line did not find a significant effect by UCMB5113 treatment, although there was a tendency for decreased number of dividing cells in inoculated samples. However, the detailed anatomy of the root tip needs to be further studied to characterize the effects at the cellular level, since the shape of the root tip is less elongated after UCMB5113 treatment. Previously, it has been shown that plant roots associated with microbes that release phytohormones affect root morphogenesis and result in overproduction of lateral roots and root hair (Persello-Cartieaux et al. 2003). IAA producing bacteria can inhibit root growth depending upon the dose of bacteria (Persello-Cartieaux et al. 2003) and such a reduced root elongation and increased shoot to root was demonstrated in sugar beet seedlings (Loper and Schroth 1986), and similar effects were reported for *Brassica juncea,* wheat, and *Arabidopsis* (Asghar et al. 2002; Khalid et al. 2004; Zamioudis et al. 2013). Auxin plays an important role in promoting cell division (Campanoni and Nick 2005), inhibits cell elongation, and increases the number of lateral roots and root hairs when in excess (Swarup et al. 2007). Our data on the *DR5:Gus* reporter line treated with UCMB5113 showed enhanced auxin response in the root cap, root meristem, and procambium of *Arabidopsis* roots. UCMB5113 was found to secrete IAA constitutively, and this production was also inducible and increased when bacteria were grown in the presence of root exudates and further stimulated by tryptophan, which has been demonstrated to be an IAA precursor in a related *Bacillus* strain (Idris et al. 2007). Treatment with a bacterial cell-free exudate on *Arabidopsis* seedlings resulted in reorientation of the main root that avoided growth towards the bacterial compounds. This demonstrates that UCMB5113 produces multiple diffusible compounds; some with auxin activity and that may interact with the auxin signaling pathways. It was demonstrated that three PGPR *Pseudomonas* strains varied in their production of auxins, where one strain that did not produce auxins still elevated *Arabidopsis* auxin responsive gene levels and resulted in a similar root phenotype as for UCMB5113 (Zamioudis et al. 2013). These results suggest that PGPR do not need to produce auxins to stimulate plant growth and that the effects on root architecture do not primarily involve PGPR auxins. Root development is a complex trait and involves multiple components, where certain transcription factors and miRNAs are some of the key regulators that may be affected as a result of PGPR action (Tian et al. 2014).

The analysis of GA in plants inoculated with UCMB5113 showed that bacteria affected the GA levels differently in roots and shoots in *Arabidopsis* seedlings after 6 days of co-cultivation compared to wild type. While total levels increased somewhat in roots after UCMB5113 treatment, the levels in shoots were almost halved. However, GA_24_, GA_34,_ and GA_15_, GA_19_, GA_24_, GA_34,_ and GA_1_, GA_8_, GA_51_, GA_53,_ and GA_15_, and GA_44_, respectively, showed increased levels in root and shoot of *Bacillus*-inoculated plants. GA production was found in rhizobacterial cultures of red pepper, where several *Bacillus* strains were found to stimulate growth and to produce different GAs (Joo et al. 2004). Our studies on *Arr5:Gus* transgenic marker with UCMB5113 enhanced expression of CK in root and indicates that CK metabolism is affected by UCMB5113 and probably involved in growth modulation. UCMB5113 could actively metabolize CKs as indicated by rapid disappearance of CK ribosides and *O*-glucosides present in the medium. At the same time, rapid increases in CK bases occurred with receptor binding properties suggesting an active role in modulation of plant roots (Spíchal 2012). The interconversion of moderately biologically active CK ribosides into highly biologically active CK bases coupled with the decreases of the inactive conjugated storage forms of CKs indicates that UCMB5113 is capable to stimulate plant growth. The ability of UCMB5113 to elevate both CK and auxin expression in roots, as reveled by use of reporter gene plants, indicates a complex effect on root growth. The key regulator SHY2, controlling meristem size and development, is stimulated by CK but repressed by auxin and also influenced by other hormones (Pacifici et al. 2015). Increased SHY2 activity due to a changed hormonal balance could be one factor that contributes to the arrested primary root growth caused by UCMB5113. The analysis of brassinosteroids showed a mixed pattern, where the level of different brassinosteroids in roots and shoot varied among the treatments. In roots, brassinolide, castasterone, and teasterone were increased by low amounts of UCMB5113, while in leaves, basal levels were generally higher and less affected but down-regulated in the case of teasterone and typhasterol. The relatively profound changes and the tissue specific patterns still point to an important effect of UCMB5113 on these hormones and a role in the observed changes in growth.

In plants, the JA signaling pathways are often activated by plant growth-promoting microbes after root colonization (Van der Ent et al. 2009; Pieterse et al. 2012). The previous studies suggests that ethylene and JA signaling pathways may interfere in auxin transport and signaling with subsequent influence on root development and architecture (Stepanova et al. 2007; Swarup et al. 2007). Our results revealed that the root structural effects of *Arabidopsis* caused by UCMB5113 are not dependent on the classical JA signaling pathway but involve other signals. The UCMB5113 total exudate fraction also caused root growth avoidance, while a lipopeptide fraction with antibiosis properties not gave these effects suggesting that the primary root inhibition effects are not due to toxic secondary metabolites but rather involves other factors. The effect on primary root growth by UCMB5113 exudate was lower than when intact bacteria were used. This can be a matter of differences in local concentration of secreted active compounds that the physical interaction with bacteria is needed or that the bacterial secretome change as a result of the plant interaction which calls for further studies.

The bacterial exudate added to roots stimulated plant growth as seen by increased leaf size and more extended root system of *Arabidopsis* Col-0 wild-type plants. The JA mutants *coi1*-*16* and *jar1* also responded by increased root growth, but only *coi1*-*16* showed increased leaf growth. Opposite effects were observed for the salicylic acid and ISR compromised *npr1* plants with increased leaf size, but no effects on root growth after addition of *Bacillus* exudates which indicates that living cells and transcriptional responses of the corresponding genes are needed for growth promotion. The UCMB5113 exudate also affected the flowering phenotype of some lines with stunted or higher inflorescence structures. In this case, *npr1* plants were more stunted, while Col-0 were higher as well as *coi1*-*16* plants, but here, a solvent effect was also observed, where methanol gave some stimulation. It has been reported that salicylic acid deficiency stimulates leaf biomass and seed production (Abreu and Munné-Bosch 2009). On the other hand, plant responses to stress by JA signaling usually restrict plant growth (Hou et al. 2013). In our case, it seems like the lipopeptide fraction has the major effect on JA compromised plants indicating that other signals than JA are stimulated by the lipopeptide fraction-stimulating growth. There is no clear correlation between effects on growth and disease suppression against *Alternaria* (data not shown) among the *Arabidopsis* lines indicating that *Bacillus* derived growth promotion does not operate through the same systems that improve stress tolerance of plants. Dependence on JA for bacterial primed ISR has been demonstrated for several other systems (Conrath 2011; Pieterse et al. 2012).

The data presented in this work provide further information of the beneficial plant–microbe interactions and impact of microorganisms on plants hormones, cellular, and root architecture development as well as the role of some signals. Exudates are complex mixtures of proteins and low molecular weight compounds with multiple functions. Root colonizing bacteria frequently form a biofilm at the rhizoplane that provide further functionality and may stimulate further interaction between the host and the microbe and also serve as a diffusion barrier (Vlamakis et al. 2013). The ability of UCMB5113 to form biosurfactants supporting biofilm formation is thus an important feature for root colonization. The interaction mechanisms in soil are unfortunately very difficult to study, but investigations using in vitro conditions with different model systems can at least approach many of the molecular mechanisms behind plant growth promotion by PGPR and support development of useful microbial tools to support crop production and more bio-based production systems using beneficial microbes as an ecosystem service.

### **Author contribution statement**

SA, SB, and JM conceived and designed research. SA, DT, JR, and ON conducted experiments. DT, JR, and ON performed the hormone analysis. DVP generated material for CK analysis. SA contributed with the other analysis. SA, DT, JR, and JM analyzed the data. SA wrote the first version of the manuscript. All authors read and approved the manuscript.

## Electronic supplementary material

Below is the link to the electronic supplementary material.

Supplementary material 1 (DOCX 1205 kb)

## Abbreviations

CK: Cytokinin
GA: Gibberellins
ISR: Induced systemic resistance
JA: Jasmonic acid
PGPR: Plant growth-promoting rhizobacteria
