# Supplementary material for: Analysis of plant growth-promoting properties of Bacillusamyloliquefaciens UCMB5113 using Arabidopsis thaliana as host plant
Source: Planta. 2016 Aug 19;245(1):15–30. doi: 10.1007/s00425-016-2580-9 (PMC5226999; doi:10.1007/s00425-016-2580-9)
Supplement: Supplementary file 1 — Supplementary material 1 (DOCX 1205 kb) [file 425_2016_2580_MOESM1_ESM.docx]

**Electronic supplementary material**

**Supplementary Fig. S1** Effect of crude lipopeptide compounds on *A*. *thaliana* seed production**.** One-week old seedlings of *A*. *thaliana* Col-0 were treated on the roots with water, 5% methanol (solvent control), or enriched lipopeptide fraction (LP) and grown on 0.5x MS agar plates and effects on seed size **(a)** and weight **(b)** determined after 48 days for *Arabidopsis* Col-0 plants and included a group (LPx2) where another LP treatment was carried out after 30 days

**Supplementary Fig. S2** Effect of crude lipopeptide compounds on growth of *A*. *thaliana* Col-0 wildtype and mutant plants. One-week old seedlings of *A*. *thaliana* Col-0 and the mutants *coi1-16*, *jar1*, *myb72* and *npr1* were treated on the roots with water, 5% methanol, or enriched lipopeptide fraction (LP) and grown on 0.5x MS agar plates and after fifteen days the seedlings were transplanted to soil and appearance observed 12 days later

**Supplementary Fig. S3** Effect of crude lipopeptide compounds on flowering of *A*. *thaliana* Col-0 wildtype and mutants**.** One-week old seedlings of *A*. *thaliana* Col-0 and the mutants *coi1-16*, *jar1*, *myb72* and *npr1* were treated on the roots with water, 5% methanol (solvent control), or enriched lipopeptide fraction (LP) and effects on flowering observed

**Supplementary Fig. 1**

**a**

| **control**  **methanol**  **LP**  **LPx2** | **** |
| --- | --- |

**b**

**Seed weight (g)**

**a**

**a**

**b**

**c**

**Treatment**

**Supplementary Fig. 2**

| ***Npr1 myb72 jar1 coi1-16* Col-0** |  |
| --- | --- |

**control methanol LP**

**Supplementary Fig. 3**

| **Col-0**  ***coi1-16***  ***jar1***  ***myb72***  ***Npr1*** | **** |
| --- | --- |
|  | **control methanol LP** |
